# Factors affecting implementation and pass rates of surgical instrument moistening

**DOI:** 10.1186/s12879-021-06471-3

**Published:** 2021-08-04

**Authors:** Yongdeng Huang, Yan Huang, Yanhua Chen, Wei Pan, Juan Hu, Liangying Yi

**Affiliations:** 1 Central Sterile Supply Department, West China Second University Hospital, Sichuan University/West China School of Nursing, Sichuan University, Chengdu, Sichuan China; 2 Key Laboratory of Birth Defects and Related Diseases of Women and Children (Sichuan University), Ministry of Education, Chengdu, Sichuan China

**Keywords:** Surgical instruments, Sterilization, Nurses, Cross infection

## Abstract

**Background:**

Moistening of surgical instruments affects the quality of instrument cleaning, thereby affecting the degree of cross-contamination and in-hospital infection among patients. Surgical instruments should be kept moist immediately after use in order to avoid concentrations of contamination remaining on surgical instrument surfaces. Implementation and pass rates of surgical instrument moistening have been rarely studied. We aimed to investigate the factors affecting implementation and pass rates of surgical instrument moistening.

**Methods:**

A cross-sectional study was conducted to investigate surgical instrument moistening procedures within 22 clinical departments of the West China Second University Hospital, Sichuan University over 122 days between September and December 2019. We collected data from departmental staff using an interviewer-administrated questionnaire. Data about implementation and pass rates of surgical instrument moistening was analyzed in SPSS20.0.

**Results:**

Implementation and pass rates of surgical instrument moistening were 57.25% and 31.98%, respectively. Factor analysis showed that implementation rates of moistening were affected by instrument structure (X^2^ = 143.670; *P* = 0.001), the number of instruments inside the pack (X^2^ = 140.135; *P* = 0.001), and the person responsible for keeping surgical instruments moist (X^2^ = 8.052; *P* = 0.005). Correlation analysis showed that instrument structure and the number of instruments inside the pack were negatively correlated with implementation rates of moistening. The more complex the structure and the greater the number of the instruments inside the pack, the lower implementation rates of moistening.

**Conclusion:**

Implementation and pass rates of surgical instrument moistening were low, and failed to meet the central sterile supply department applicable industrial standard, hence the potential risk of hospital-acquired infection was considerable. Staff that manipulate reusable surgical instruments should be trained to properly moisten the instruments and institutional protocols should be established to ensure standardization and respect of guidelines.

## Background

Thorough cleaning of surgical instruments is vital to their disinfection and sterilization [1]. However, the efficiency and degree of sterilization depend to a large extent on logistics and sterilization management procedures, namely surgical instrument transportation, storage and cleaning procedures. There is the risk of surgical instruments not being cleaned properly or immediately after use, including insufficient moistening, which results in potentially hazardous concentrations of contamination remaining on surgical instrument surfaces, leading to cross-contamination. Incomplete cleaning of instruments is one form of poor sterilization which contributes to hospital-acquired infections. In a landmark study of sterilization procedures in the central sterile supply department (CSSD), Liu and Liang (2013) stated that surgical site infections accounted for 14–16% of hospital-acquired infections among hospitalized patients, and that approximately 20% of surgical site infections were ascribed to poor instrument hygiene quality [2]. According to the Central Sterile Supply Department (CSSD)’s directive (transl.)—*Part 2: Standard for* *Operating Procedure of Cleaning, Disinfection and Sterilization* (WS 310.2-2016) [3], “The user shall timeously remove visible contaminants from diagnostic and treatment instruments, apparatus and articles after use, and moisten them according to actual situations.”

There have been numerous studies investigating methods for keeping surgical instruments moist and effects of moistening, but factors affecting implementation and pass rates of surgical instrument moistening have been rarely studied. This case study considers the factors in relation to moistening of surgical instruments which affects the quality of instrument cleaning, and thereby affecting the degree of cross-contamination and in-hospital infection among patients, which should be monitored by medical staff and hospital management in their efforts to lower infection and contamination levels.

## Methods

### Study setting

A survey was conducted within 22 clinical departments of the West China Second University Hospital, Sichuan University over 122 days between September and December 2019. The 22 departments in the hospital rely on reusable surgical instruments; other departments in the hospital used disposable surgical instruments or did not use surgical instruments. The 22 departments have a total of 1580 hospital beds, and the average number of operations performed across the 22 departments per year is around 70,000.

### Survey tools

We designed an interviewer-administrated questionnaire based on the aforementioned CSSD directive (*Part 2: Standard for* *Operating Procedure of Cleaning, Disinfection and Sterilization* (WS 310.2-2016)) [3] in order to investigate instrument moistening procedures in the hospital. We pre-tested the questionnaire to assess its reliability and validity before using it to collect data. Cronbach’s alpha for the questionnaire was 0.911 and test–retest reliability coefficient was 0.825. The questionnaire which had high reliability and validity was composed of 4 parts:

Part 1: Consisted of 7 questions, and was used to collect data about which campus and which department each respondent worked in, as well as the respondent’s name, age, length of service, educational background, and job title.

Part 2: Consisted of 4 open-ended questions regarding surgical procedure names, a description of the relevant surgical pack, the number of instruments inside the pack, the end date and end time of surgery, and time of moistening; these were followed by 11 closed-ended questions regarding place of moistening, the person responsible for moistening, moistening method, moisturizing liquid/agent, and effects of moistening on different instruments.

Part 3 and Part 4: Each of these questionnaire parts contained 1 question. Each respondent was asked to detail reasons why moistening procedures might fail (12 possible answers), and (in Part 4) provide reasons why instruments were not moistened (7 possible answers). Each respondent was allowed to choose 1 or more answers to each of the 2 questions. However, the respondent was not required to answer the questions in Part 3 and 4 if instruments were moistened in compliance with the CSSD standard.

The questionnaires were administrated by us who had received training on helping the respondents fill in questionnaires and also possessed experience of conducting on-site investigations for keeping surgical instruments moist. We visited the 22 departments to investigate the following: whether surgical instruments were being kept moist after use; type of moistening agent used; place and time of moistening; method for keeping instruments moist; and, effects of moistening on different instruments. A sample of nurses and other hospital workers were asked to explain why moistening procedures might fail, and specify reasons why the instruments were not being kept moist.

We have classified reusable surgical instruments into three categories: common instruments, instruments with articulation joints or grooves, and cannulated instruments. We recorded information concerning the effects of moistening of instruments in different surgical packs, and calculated pass rates of moistening for the three categories of instruments and those for surgical packs containing different numbers of instruments. We also collected information concerning the effects of moistening handled by different persons (nurses or workers). We have supplemented the information provided by respondents by taking photos and recording videos demonstrating moistening procedures, for the purpose of further analysis and discussion by the research team.

Moistening is considered adequate when moisturizing agent is sprayed on the instruments immediately after use; when the surface, articulation joints and grooves of the instruments are fully and evenly covered with moisturizing gel; and when they are kept moist right up to the moment of delivery to CSSD for subsequent processing [4]. We found that Conbizyme instrument moisturizing gel is used in the hospital. Conbizyme is the brand name of the moisturizing gel produced by Nanjing Jusha Commercial & Trading Co., Ltd. in China; it is a ready-to-use instrument moisturizing product which is sprayed directly without dilution on instruments at a distance of around 10 cm.

Three are three spray methods: *Random spray* is defined as discretionary spray, the degree of which varies according to moistening handler’s own convenience without regard to the effect of moistening. In the ‘*Evenly*’ *spray* method, the instrument surface is evenly covered with moisturizing agent in order to effectively moisten the instrument. With the *Targeted spray* method, the instruments with their different structures from different angles are sprayed in such a way to achieve complete moistening. We took notes of the respective spray methods during the on-site inspection.

All research methods were carried out in accordance with the relevant guidelines and regulations. This study was conducted in accordance with the Declaration of Helsinki, and was approved by the Medical Ethics Committee of West China Second University Hospital, Sichuan University (No.: YXKY2020LSP(171)). Informed consent for participation was obtained from all nurses and workers targeted by this study. During our on-site investigation, we presented our work-related identity cards to the nurses and workers of the 22 departments, orally explained the purpose and significance of this study to them, and informed them that they had the right to refuse to answer our questions or withdraw from the study at any time. We then allowed them to choose whether to participate in the study. After obtaining their verbal consent to participate in this study, we verbally presented them with the questionnaire items, and then recorded their responses to the questionnaire items. The whole interview process, including our oral presentation and obtaining participants’ verbal consents, was witnessed by the head nurse of their respective department. The Medical Ethics Committee of West China Second University Hospital, Sichuan University reviewed and approved the research proposal and procedure of verbal consent of this study, and felt that we did not need to obtain written consent from the participants.

### Statistical methods

We used MS Excel 2007 for data entry, and analyzed data using the SPSS20.0 statistical package. We applied the Pareto principle in our analysis of factors, i.e. whether the number of significant factors is greater than that of non-significant factors [5]. The data are expressed as percentages, frequency and relative frequency. In addition, the Chi-square test and rank sum test are used to compare factors, and the degree of correlation between variables has been analyzed by applying the rank correlation approach. Bivariable test results show factors affecting pass rates of moistening to be statistically significant at the (*P* < 0.05) level.

## Results

### Implementation of surgical instrument moistening procedures

It was found that only reusable surgical instruments from 3 (13.64%) of the 22 departments were moistened. A total of 45,717 reusable surgical instruments from the 22 departments were investigated, among which 26,172 instruments (implementation rate = 57.25%) were kept moist, and 8369 instruments (pass rate = 31.98%) were moistened in compliance with the CSSD standard. The implementation and pass rates of surgical instrument moistening were low, as shown in Table 1.

**Table 1:** Implementation of surgical instrument moistening in each department

Departments	Number of surgical instruments (%)	Number of instruments kept moist	Implementation rate of moistening (%)	Number of instruments moistened in compliance with CSSD standard	Pass rate of moistening (%)
Inpatient operating rooms	27,105 (59.29)	21,506	79.34	6139	28.55
Delivery rooms	12,611 (27.59)	0	0	–	–
Outpatient operating rooms	4097 (8.96)	4097	100	2152	52.53
In vitro fertilisation	818 (1.79)	0	0	–	–
Pediatric Cardiology	328 (0.72)	0	0	–	–
Radiology	569 (1.24)	569	100	78	13.71
Other clinical departments	189 (0.41)	0	0	–	–
Total	45,717	26,172	57.25	8369	31.98

### Reasons why surgical instruments were not being kept moist

**Fig. 1:**
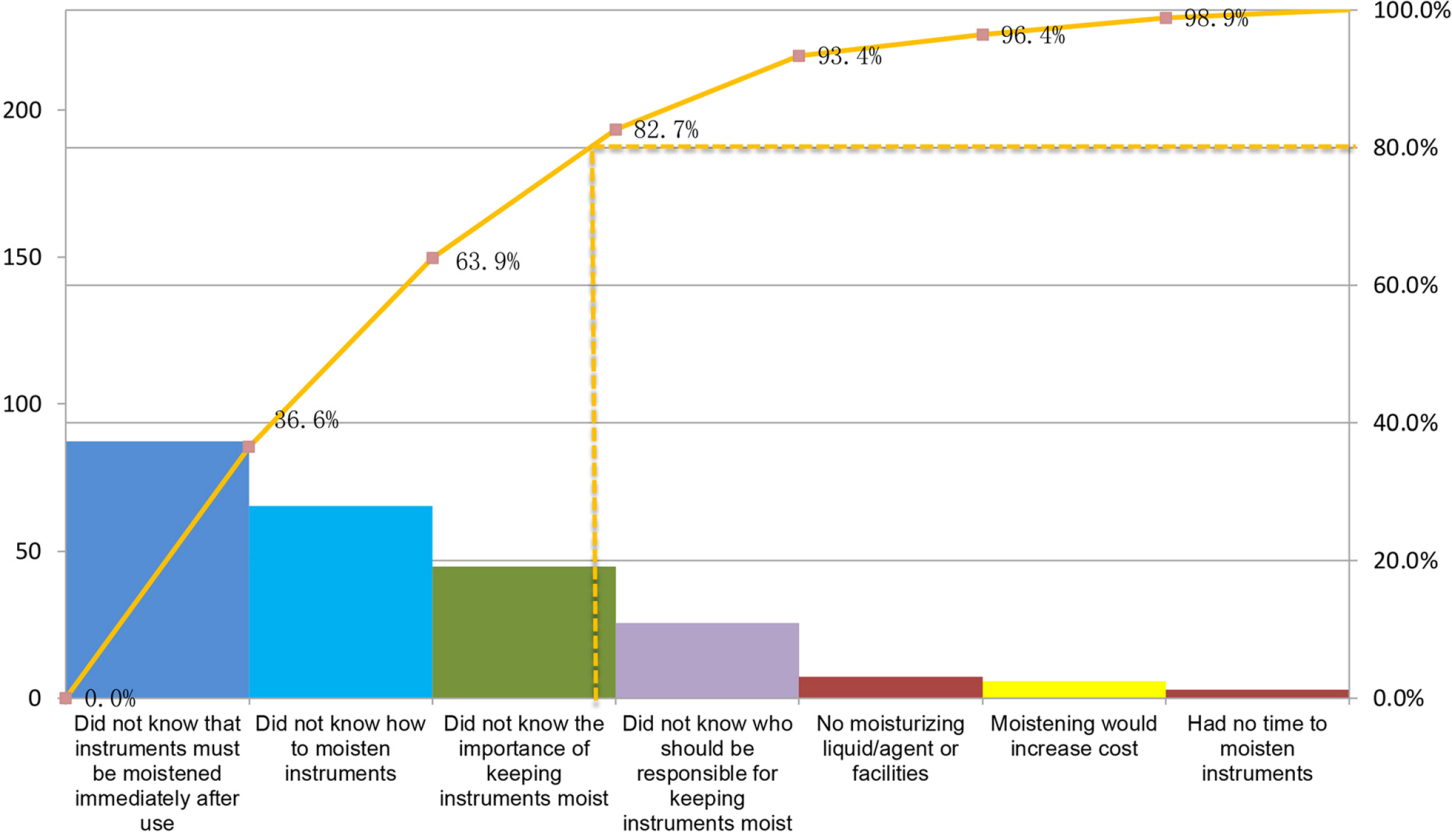
Reasons why surgical instruments were not being kept moist

The Pareto diagram has been utilized to analyze the reasons why surgical instruments were not being kept moist. Our analysis showed that the main reasons included “did not know that instruments must be moistened immediately after use”, “did not know how to moisten instruments”, and “did not know the importance of keeping instruments moist”, as shown in Fig. 1.

### Reasons why moistening procedures failed or were insufficient

**Fig. 2:**
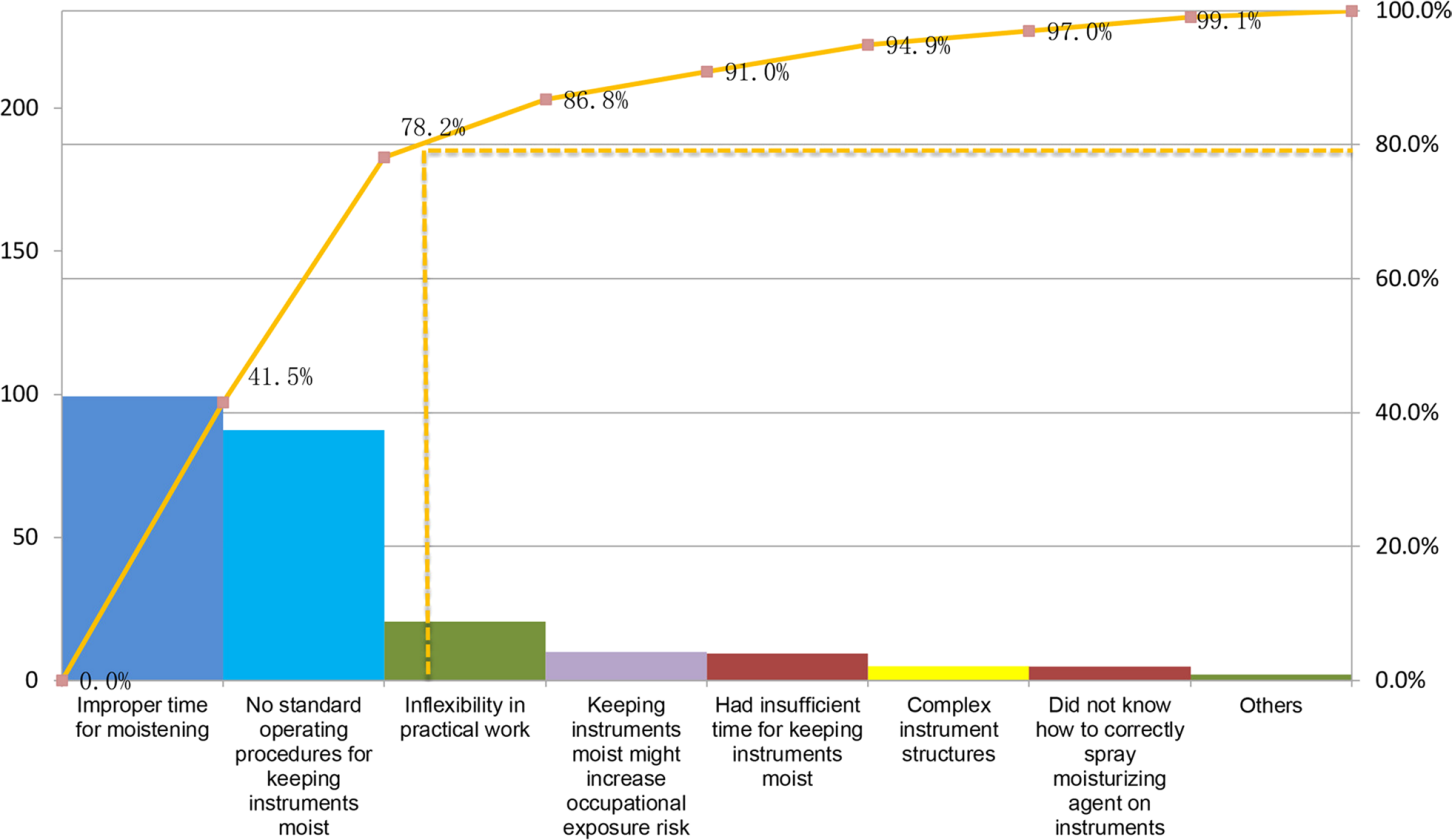
Reasons why moistening procedures failed or were insufficient

The Pareto diagram has been adopted in order to analyze the reasons behind failures in moistening procedures or coverage. Reasons include improper time for moistening, and lack of standard operating procedures for keeping instruments moist, as shown in Fig. 2.

### Time of moistening (interval between end of instrument use and start of moistening)

Among the three departments which moistened instruments, the interval between end of instrument use and start of moistening among the outpatient operating rooms lasted 4.04 min on average (4.001 < 95% confidence interval < 4.079); that of the inpatient operating rooms lasted 4.88 min on average (4.862 < 95% confidence interval < 4.898); and, that of the radiology department lasted 172.5 min on average (172.169 < 95% confidence interval < 172.831).

### Spraying methods

It was found that the percentages of instruments subject to random spray, evenly spray, and targeted spray were 83.56%, 12.33%, and 4.11%, respectively.

### Factors affecting keeping surgical instruments moist

Pass rates of moistening were calculated for instruments with different structures, surgical packs containing different numbers of instruments, and instruments moistened by different staff members. Single factor analysis was conducted using the Chi-square test, the results of which show that instrument structure, the number of instruments inside the pack, and the person responsible for keeping instruments moist, constitute the main factors affecting the pass rates, as shown in Table 2.

**Table 2:** Single factor analysis on factors affecting keeping surgical instruments moist

Item	Number of instruments kept moist (%)	Number of instruments moistened in compliance with CSSD standard	Pass rate of moistening (%)	X^2^	*P* value
Instrument structure				143.670	0.001
Common instruments	7852 (30.00)	4776	60.83		
Instruments with articulation joints or grooves	15,703 (60.00)	3305	21.05		
Cannulated instruments	2617 (10.00)	288	1.10		
The number of instruments inside the pack				140.135	0.001
< 10 pcs	1581 (6.04)	1316	83.24		
10–20 pcs	2577 (9.85)	1733	67.25		
21–30 pcs	2018 (7.71)	875	43.36		
31–40 pcs	781 (2.98)	174	22.28		
> 40 pcs	250 (0.96)	33	13.20		
Person responsible for keeping instruments moist				8.052	0.005
Nurses	18,320 (70.00)	7402	40.40		
Workers	7852 (30.00)	967	12.32		

Correlation analysis was conducted for the factors affecting moistening of surgical instruments. The results show that the more complex the structure and the greater the number of the instruments inside the pack, the lower the pass rates of moistening; the results also show that the pass rates among nurses was higher than that among workers, as detailed in Table 3.

**Table 3:** Correlation analysis on factors affecting keeping surgical instruments moist

Item	Correlation coefficient	*P* value
Instrument structure	− 0.525	0.001
The number of instruments inside the pack	− 0.203	0.001
Person responsible for keeping instruments moist	0.194	0.001

## Discussion

This study shows that the implementation rate for the sample of surgical instruments in the hospital was only 57.25%. The main reasons for this were that many of the nurses and workers of the clinical departments were not fully knowledgeable of the methods or importance of keeping surgical instruments moist. It is evident that many of the nurses and workers are in need of more training as regards to keeping surgical instruments moist and reducing the risk of cross-contamination. The low pass rates are also a reflection of a lack of standard operating procedures for keeping instruments moist, and the need for harmonization of guidelines. As reported by other scholars in the field, contaminants should be removed from the reusable surgical instruments as soon as possible following use and sent to CSSD within 30 min in order to minimize the risk of cross-contamination; in short, the instruments should be kept moist [6]. Numerous dried contaminants on the instruments can be difficult to remove, will increase the cost of cleaning, and might even cause corrosion, which impairs instrument function and shorten the service life of instruments, and thus increases the average unit cost [4]. Therefore, surgical instruments should be kept moist immediately after use if they are unavailable for timely cleaning. It has been demonstrated in some studies that contaminants would dry within several minutes if not processed immediately after use [7]; bacteria could multiply in 4–20 min on dried contaminants, forming a biofilm within 2 h [8]. The earlier surgical instruments are moistened, the better the moistening effect is. It is therefore recommended that surgical instruments should be kept moist immediately following surgery [9].

According to the correlation analysis, the main factors influencing the moistening of surgical instruments include instrument structure, the number of instruments inside the pack, and the person responsible for keeping instruments moist. The more complex the instrument structure, the less effective the moistening; therefore, special methods should be used for instruments with more complex structures. Ultrasonic scalpels, connecting cables for argon beam coagulation, among other instruments with complex structures, should be kept moist via separate multienzyme soaking [10]. Appropriate spraying methods should be adopted in order to ensure sufficient moistening, e.g. unlock instrument articulation joints prior to spraying, and carrying out targeted spray on articulation joints, grooves and cannulations of instruments [9], and place instruments in the recycling box for spraying when there are many instruments inside the pack. Pass rates among workers were significantly lower than those among the nurses, possibly because the workers’ primary responsibility was to collect and deliver contaminated instruments, and because most of them only had secondary education qualifications, and lacked medical knowledge concerning the importance of moistening. Ideally, surgical instruments should be moistened by nurses as they have more professional knowledge and higher sense of responsibility.

## Conclusion

It was found in this case study that implementation and pass rates of surgical instrument moistening were low, and failed to meet the CSSD applicable industrial standard, hence the potential risk of hospital-acquired infection was considerable. Staff that manipulate reusable surgical instruments should be trained to properly moisten the instruments and institutional protocols should be established to ensure standardization and respect of guidelines.

## Data Availability

The datasets used and/or analyzed during the current study are available from the corresponding authors on reasonable request.

## Abbreviation

CSSD: Central sterile supply department
